# Saltwater intrusion indirectly intensifies *Phragmites australis* invasion via alteration of soil microbes

**DOI:** 10.1038/s41598-022-20555-3

**Published:** 2022-10-04

**Authors:** Carolyn S. Schroeder, Nelle K. Kulick, Emily C. Farrer

**Affiliations:** 1 Environmental Protection Agency, 302 12th St NW, Washington, DC 20004 USA; 2 Department of Anthropology, Tulane University, 6823 St. Charles Avenue, New Orleans, LA 70118 USA; 3 Department of Ecology and Evolutionary Biology, Tulane University, 6823 St. Charles Avenue, New Orleans, LA 70118 USA

**Keywords:** Climate-change ecology, Community ecology, Invasive species, Microbial ecology, Wetlands ecology

## Abstract

Although global change clearly influences species invasion, the exact mechanisms by which global change either intensifies or limits invasive spread remain elusive. Global change can affect invasion directly by altering abiotic conditions, as well as indirectly by altering the abundance and composition of interacting species. Here we examine the relative impacts of direct effects of saltwater intrusion and indirect effects via microbial interactions on the expansion of a model invasive plant species, *Phragmites australis*, in freshwater marshes of coastal Louisiana. Using a mesocosm experiment, we found that overall salinity strongly increases invasion, but the direction and magnitude of direct and indirect effects were context dependent. Indirect effects of salinity, via alterations in soil microbial composition, increased invasive performance when grown in monoculture and decreased native performance in native-only communities. However, when *P. australis* and natives were grown together, microbial indirect effects were not important; rather the salinity treatment increased *P. australis* invasion through reduction of native plant growth. Results suggest that salinity-induced alteration of soil microbes will increase susceptibility of native communities to invasion and promote *P. australis* monoculture expansion in later stages of invasion; whereas non-microbial effects of salinity are more important in early stages of invasion when *P. australis* is competing with native species. More broadly, these results underscore the importance of considering microbially-mediated indirect effects of global change in investigating the long-term outcomes of plant species interactions.

## Introduction

Global environmental change is dramatically altering many ecological processes, including plant species invasion^1,2^. Although global change is expected to affect invasion dynamics, ecologists have an incomplete understanding of whether global change will intensify or limit invasive spread and the mechanisms underlying these outcomes. Species responses to global change are governed by mechanisms that fall into two primary categories: direct and indirect effects^3–8^. Direct effects refer to the influence of abiotic factors per se on a species’ growth or survival, whereas indirect effects refer to impacts of abiotic factors that are mediated by changes in co-occurring species. A growing body of literature indicates that indirect effects of global change can be just as strong, if not stronger, than direct effects^6,9–11^. Distinguishing when direct vs. indirect effects dominate is important for determining when explicitly modeling species interactions is necessary for forecasting ecosystem response to global change and can aid in developing effective management strategies.

Shifting abiotic conditions can impact plant species indirectly via changes in distributions of interacting plant species^10^ and also by alteration of interactions among species at different trophic levels. For example, global change-initiated dissonance between plants and their pollinators and herbivores has been relatively well-documented^3,9,12^. Impacts on plant–microbe interactions have received less attention (but see^13–15^). Previous studies have investigated how plants mediate soil biota responses to shifts in drivers of global change^13,16,17^ and the effects of altered abiotic conditions on microbe-microbe interactions^18^. However, whether soil biota composition mediates plant community responses to abiotic changes associated with global change remains largely unexplored. Soil microbial communities contain plant pathogens and mutualists that drive patterns of plant dominance and species coexistence^19^, and they are gaining increasing attention as key drivers of invasion^20,21^. Quantifying this novel type of indirect effect provides insight into mechanisms that may shape outcomes of invasion as global change progresses.

Plant invasion is a dynamic, multi-stage process that proceeds from an uninvaded native community, to initial establishment when the invader is relatively rare, to dominance when the invader is surrounded by conspecifics. Little is known about how community context may affect the relative importance of direct and indirect mechanisms. Previous work has shown that the intensity of indirect effects can depend on the attributes and abundances of interacting species^4^. It has also been shown that the strength of plant–microbe interactions is altered by the identity and density of neighboring species^22^. Thus, it is likely that areas with different levels of invasion may experience different degrees of direct and indirect effects. Understanding the relative strength of direct and indirect mechanisms at different stages of invasion can inform both invasion theory and targeted management strategies.

Elevated soil salinity is one driver of global change that will have widespread consequences for plant invasion dynamics. Soil salinization is expected to increase in many ecosystems worldwide due to increased evaporation in arid regions^23^ and exacerbation of saltwater intrusion by relative sea level rise in coastal habitats^24^. In freshwater marshes, saltwater intrusion drastically alters plant species composition^25–27^, which can have dire consequences for biodiversity, marsh integrity, and ecological function^28,29^. Saltwater intrusion also has profound impacts on soil microbial structure and function^30,31^. Thus, elevated salinity will not only have direct impacts on plant growth, but also will likely influence plant growth indirectly, via alteration of soil microbial composition.

Here, we examine the indirect effects of elevated salinity on invasion by *Phragmites australis* in freshwater marshes in coastal Louisiana. *Phragmites australis* is a globally distributed dominant marsh grass that is considered a model species for plant invasion research^32^. Using experimental mesocosms in a greenhouse setting, we test two hypotheses: (1) indirect effects of elevated salinity, via alteration of soil microbes, will be stronger than direct effects on *P. australis* and native growth, and (2) the strength of direct and indirect effects will depend on community context (level of invasion). We test direct and indirect effects in three community contexts: invasive monoculture, native community, and mixed invasive/native community. We implement an elevated salinity treatment to test direct effects of saltwater intrusion. We define microbial indirect effects as changes in plant growth that are due to salinity-induced alteration of soil microbial composition. To experimentally test microbial indirect effects of salinity, we compared the biomass of plants grown in soil inoculum from a freshwater site and soil inoculum from a saline site.

## Results and discussion

### Hypothesis 1: Indirect effects of elevated salinity, via alteration of soil microbes, will be stronger than direct effects on *P. australis* and native growth

**Figure 1:**
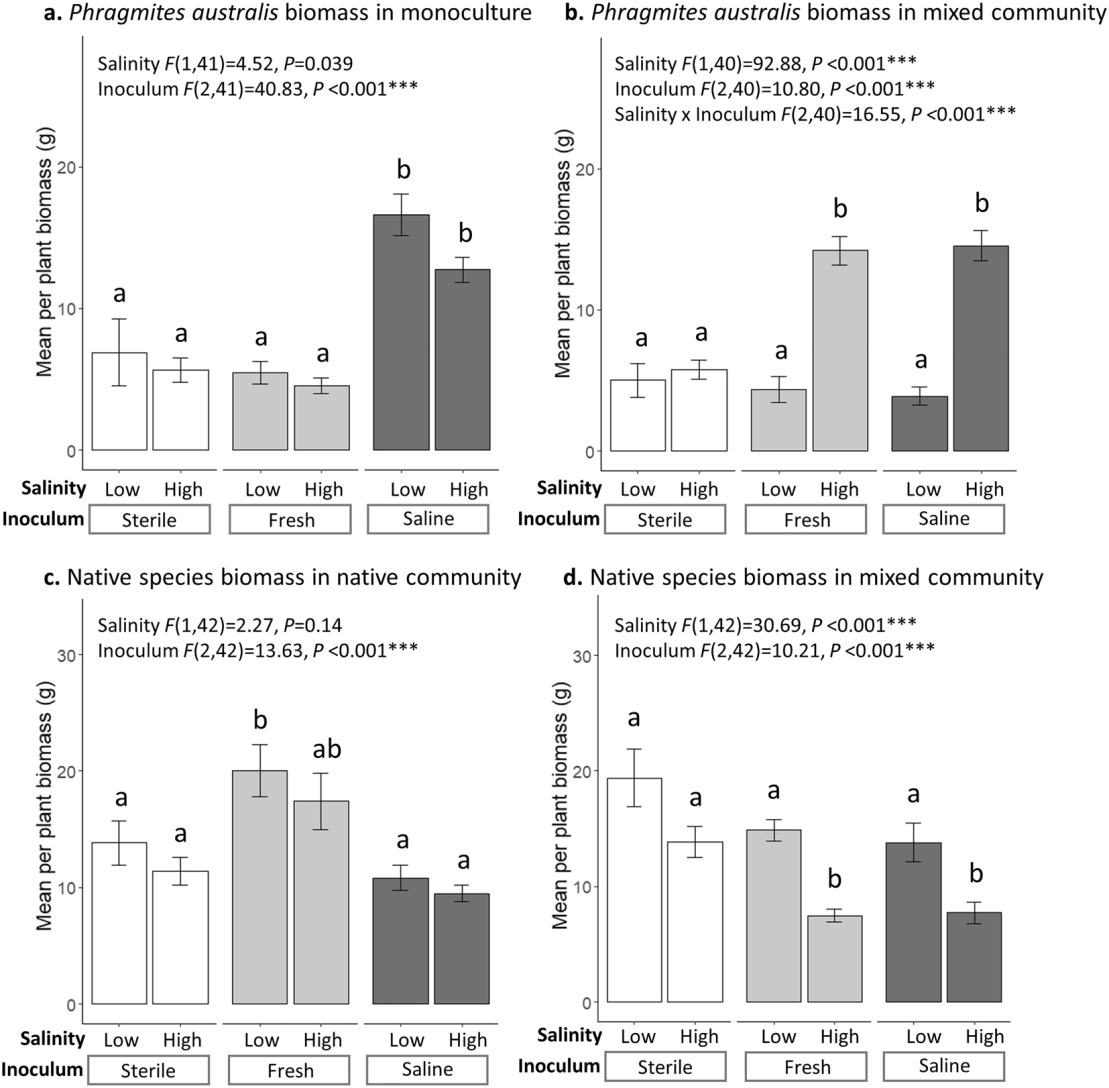
Effects of salinity (low and high) and inoculum (sterile, freshwater, and saline) on *P. australis* biomass in monoculture (**a**) and mixed communities (**b**) and on pooled native biomass in native communities (**c**) and mixed communities (**d**). White bars represent sterile soils, light grey bars represent freshwater inoculum treatments, and dark grey bars represent saline inoculum treatments. Error bars represent 1 standard error of treatment means. Two-way ANOVA results are presented. The salinity*inoculum interaction term was included in models if significant. Letters over the bars represent differences in means from Tukey post hoc tests. To visually compare biomass in different community types, total pooled biomass was adjusted to represent biomass per plant. For *P. australis,* total biomass was divided by six in monocultures and by three in mixed communities. For native species, total native biomass was divided by six in native communities, and by three in mixed communities.

Salinity had a significant negative impact on *P. australis* growth in monoculture, but the effects of saline microbial inoculum were much stronger and were positive (Table 1, Fig. 1a). Furthermore, *P. australis* growth in freshwater microbial inoculum was no different from growth in sterile soil (Fig. 1a). This indicates that salinity-induced shifts in microbial composition will promote invasion with global change. Consistent with these findings, it has been shown that endophytes from *P. australis* in saline areas had more growth promotion ability compared to endophytes from fresher areas, enhancing invader growth in saline habitats^33^. Gonzales et al. 2020 also found that dark septate endophytes improved *P. australis* seedling survival and aboveground biomass in response to salinity stress^34^. Farrer et al. 2021 observed that *P. australis* invasion had a greater impact on plant and microbial community composition in more saline marshes, compared to freshwater marshes^35^. Taken together, these findings suggest that *P. australis* may be involved in reciprocal feedbacks with soil microbiota that will be particularly beneficial in marsh systems experiencing saltwater intrusion. Previous work has shown that invasive species can benefit from positive plant-soil feedbacks that promote their expansion^36–38^, but thus far little work has assessed how abiotic variables will impact these interactions (but see^22,39^). This research underscores the abiotic context dependency of invader-microbe relationships and suggests that elevated salinity may increase the frequency of positive plant–microbe interactions that promote *P. australis* invasion.

**Table 1:** Two-way ANOVA results testing the effects of salinity (direct effects) and microbial inoculum (indirect effects) on *P. australis* biomass in monoculture and mixed communities and on pooled native biomass in native communities and mixed communities.

	Community	Salinity			Inoculum		
		*F*	*df*	*P*	*F*	*df*	*P*
*P. australis*	Monoculture	46.40	1.29	0.017*	91.93	1.29	< 0.001***
	Mixed	127.47	1.29	< 0.001***	0.0038	1.29	0.95
Native	Native	1.29	1.29	0.27	24.19	1.29	< 0.001***
	Mixed	38.72	1.29	< 0.001***	0.15	1.29	0.70

The salinity*inoculum interaction terms were not significant, so they were removed from final models. Note that analyses in this table only include two inoculum types (microbes from freshwater or saline conditions) so that they represent indirect effects, whereas analyses shown in Fig. 1 include three inoculum types (sterile, freshwater, and saline).

**Figure 2:**
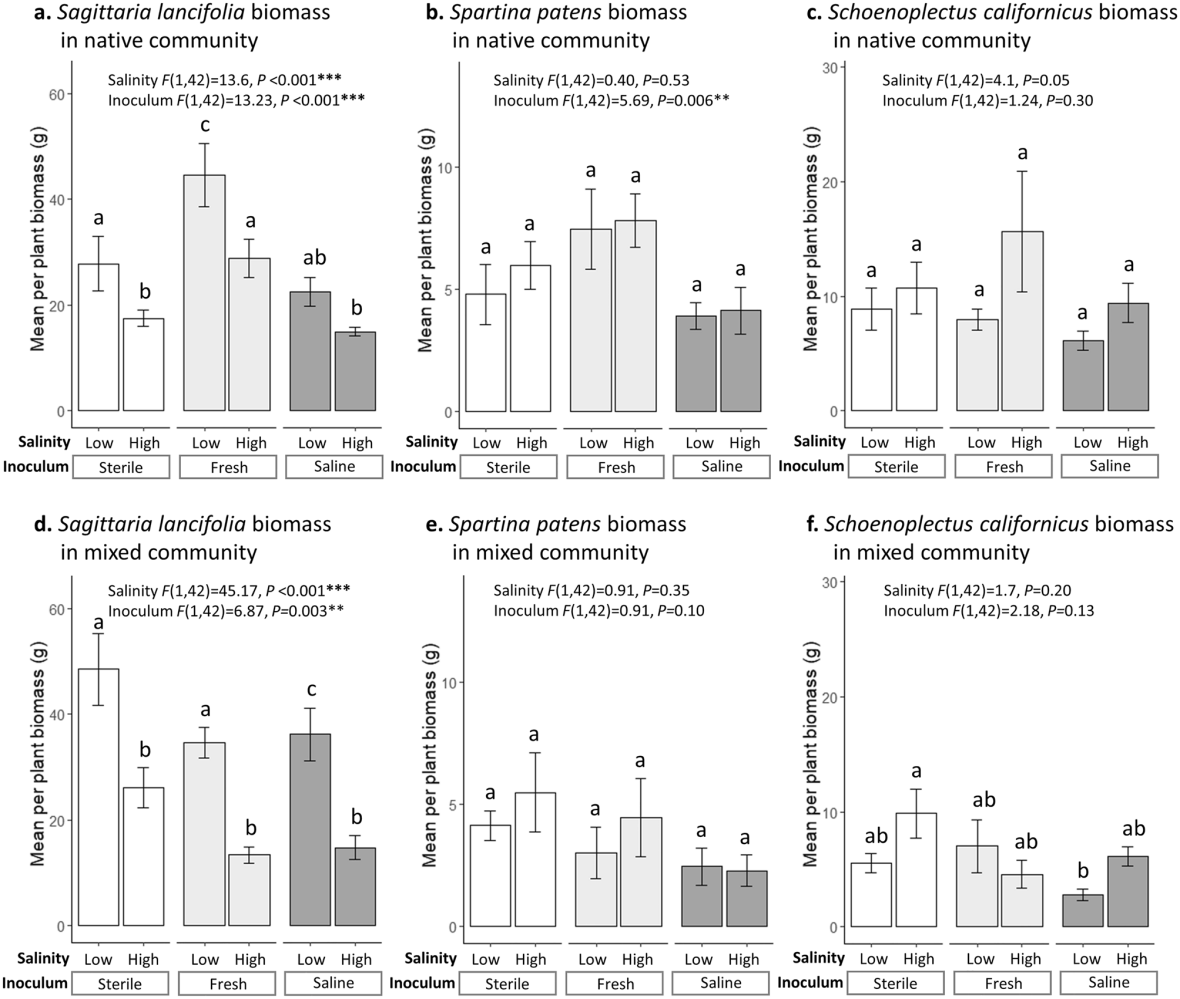
Effects of salinity (low and high) and inoculum (sterile, freshwater, and saline) on native species biomass in native communities (**a**–**c**) and mixed communities (**d**–**f**). White bars represent sterile soils, light grey bars represent freshwater inoculum treatments, and dark grey bars represent saline inoculum treatments. Error bars represent 1 standard error of treatment means. Two-way ANOVA results are presented. The salinity*inoculum interaction term was not significant, so it was not included in final models. Letters over the bars represent differences in means from Tukey post hoc tests. To compare biomass in different community types, total pooled biomass was adjusted to represent biomass per plant. There were two plants per species in the native communities, so total biomass per species was divided by two. There was only one plant per species in the mixed communities, so it was not adjusted.

The indirect effect of microbial inoculum was also stronger than the direct effect of salinity in native communities. In sharp contrast to the positive effects of saline microbes on *P. australis* growth, native growth in the native community exhibited a strong positive response to freshwater microbes (compared to sterile and saline inoculum which did not differ) (Table 1, Fig. 1c). Salinity did not directly decrease total biomass in native communities (Table 1, Fig. 1c), but it did strongly reduce growth of one of the three species, *Sagittaria lancifolia* (Fig. 2a). The strong positive freshwater inoculum effect on native growth suggests that the native species in this system form mutualisms with microbes in freshwater soils that promote their growth. Natives likely benefit from a “sympatric advantage” when growing with microbes in a low-salinity environment where they have evolved^40^. Previous work has shown that plant–microbe mutualisms tend to be stronger when they are comprised of potentially co-adapted plant hosts and soil biota that originate from the same habitat or location^40–43^. Saltwater intrusion into freshwater marshes, or indeed any type of global change that impacts microbes in an ecosystem^44–46^, will likely disrupt key plant–microbe partnerships and shift native communities into a state that is more prone to invasion.

### Hypothesis 2: The strength of direct and indirect effects will depend on community context (level of invasion)

Work in other systems^22,45,46^ and in coastal marshes^47^ has found that neighbor identity can alter the strength of plant–microbe feedbacks. However, little previous work has investigated how plant community context can affect the way in which plants respond to microbes that have shifted due to climate change (but see^39^). Consistent with our predictions, we found that the strength of microbial inoculum on *P. australis* and native growth depended on community context. While saline microbes strongly increased *P. australis* growth in monoculture (Table 1, Fig. 1a), there was no signal of microbial inoculum effects when *P. australis* was grown with native species (Table 1, Fig. 1b). We posit that *P. australis* individuals in monoculture experienced a positive plant-soil feedback involving mutualists in saline soils, the effects of which were overwhelmed by competition in mixed communities. This aligns with previous work^48^, which found that an invasive species exhibited positive plant-soil feedback, but this effect was absent when the invader was competing with native species. Correspondingly, freshwater soil inoculum had a mutualistic effect on native species in native pots (Table 1, Fig. 1c), but this effect was muted in mixed communities (Table 1, Fig. 1d). The lack of soil inoculum effects in mixed communities underscores the importance of testing soil origin effects with competition treatments. Results from monoculture and native communities alone would have led us to hypothesize that saline soil inoculum would enhance *P. australis’* ability to compete with natives. However, we found that the competition with natives masked the benefits that *P. australis* received from saline microbes. Our results imply that monoculture-forming invaders like *P. australis* are more likely to be influenced by salinity-induced changes in microbial composition after establishing dominance on the landscape.

While the direct effects of salinity on plant growth in monoculture and native communities were weak, in mixed communities salinity strongly reduced native growth (Table 1, Fig. 1d) and increased *P. australis* growth (Table 1, Fig. 1b). It is well known that elevated salinity can alter competitive dynamics among marsh plants; in other words, there can be indirect effects of salinity through altered competition^28,49^. *Sagittaria lancifolia* was the primary driver of community responses to salinity in native and mixed communities, because it produced the most biomass of the three native species and was most sensitive to salinity. It is likely that salinity and competition with *P. australis* synergistically reduced *S. lancifolia* growth in mixture (Fig. 1d, Fig. 2d), which allowed *P. australis* growth to increase in high salinity conditions (Fig. 1b). *P. australis* is known to be highly competitive via resource competition and allelopathic effects^50^ and is also known to have a high salinity tolerance^51^. It is also possible that the presence of native species facilitated *P. australis* growth in high salinity conditions (similarly to facilitation observed in high salinity conditions in^49^). These results indicate that direct effects of salinity (and corresponding changes in competitive interactions) may be more important in early stages of invasion, when the invader exists in low abundance and is surrounded by native heterospecifics.

## Conclusions

Indirect effects complicate our ability to predict the long-term trajectory of invasive populations with global change. In certain contexts, direct effects dominate plant community responses to abiotic variation^7^. However, in many cases, indirect effects resulting from interactions with co-occurring species can either neutralize, exacerbate, or counteract direct effects^6,9–11^. Thus, impacts of abiotic stressors on the direction of invasion depend on the relative strength of these direct and indirect mechanisms. We found that although direct effects of salinity slightly decreased *P. australis* growth in monoculture, indirect effects via alteration of soil microbes created a net positive impact on *P. australis* growth. However, this indirect effect only increased *P. australis* growth in monoculture and had no effect when *P. australis* was in competition with native species. Similarly, microbial indirect effects strongly reduced native growth in native communities, but had little impact when natives were grown with *P. australis.* Our results imply that the relative impacts of direct and indirect effects of salinity differ across stages of invasion. Microbial indirect effects may make native communities more vulnerable to invasion, and also maintain *P. australis* dominance once it has established monocultures. Non-microbial effects of salinity may be more important in early stages of invasion when the reduction in competition from native species at high salinity promotes *P. australis*. This work demonstrates the need to consider indirect effects of shifting abiotic variables when predicting species responses to global change.

## Methods

We used a factorial greenhouse experiment to quantify the direct effects of salinity and the indirect effects of salinity via microbes on invasive and native performance. Three different community types were included in this experiment: invasive monoculture, native community, and a mixed invasive and native community. Invasive monocultures consisted of six *P. australis* individuals. The native communities comprised two individuals each of three native species dominant in freshwater (0-3ppt salinity) and intermediate marshes (2–6 ppt salinity): *Sagittaria lancifolia, Schoenoplectus californicus,* and *Spartina patens,* for a total of six individuals per mesocosm^52^*.* Mixed communities contained three *P. australis* individuals and one of each native species. This experimental design is similar to a De Witt-type replacement series, where density is held constant^53^.

Effects of salinity were tested with a salinity addition treatment of low (0.19 ppt +/− 0.01 SE) and high (14.75 ppt +/− 1.6 SE). Direct effects of salinity are assessed by comparing the growth of natives in native communities in low vs. high salinity, and the growth of *P. australis* in monoculture in low vs. high salinity. Due to competition between *P. australis* and native plants in mixed pots (and the potential for competitive indirect effects), we did not interpret the salinity treatment in mixed pots to represent direct effects. We recognize that competition is also occurring among the three native species in the native community pots, but here we treat natives as one entity, distinct from the invader. Indirect effects of salinity via changes in the microbial community (microbial indirect effects) were tested using soil microbial inoculum from a freshwater and a saline site. To assess the overall effects of inoculum on plant growth, we also included a sterile soil treatment. Each of the treatment combinations (low/high salinity, fresh/saline) were replicated eight times in the three community types, for a total of 144 mesocosms.

Plants were grown from rhizomes that had been trimmed of fine roots, standardized to two nodes, and surface-sterilized in 10% bleach for 5 min^38^. After surface-sterilizing, rhizomes were sprouted in deionized water before planting. *Phragmites australis* (European haplotype, M) and *S. lancifolia* rhizomes were collected from local marshes (Pass A Loutre State Wildlife Management Area, Venice, LA, and Turtle Cove Research Station, Manchac, LA). We obtained *S. californicus* and *S. patens* rhizomes from a local plant nursery (TBeb Wetland Nursery, Montegut, LA). Collection of plant material complied with relevant institutional, national, and international guidelines and legislation.

Abiotic mesocosm conditions were designed to approximate field conditions in marshes in coastal Louisiana. Each mesocosm consisted of a five-gallon bucket, which was placed inside another bucket filled with water, ensuring that soils remained saturated. Sterilized 5-gallon buckets were filled with a 3:1 mixture of river sand and sphagnum moss potting medium. Potting medium was autoclaved at 134 °C at 100 kPA for 60 min^47^. Pots were consistently flooded by automatic drip irrigation with deionized water. For the direct effect treatment, saline water (500 mL, 20ppt) was added three times a week (in the late afternoon) to the high salinity treatment pots, while the same amount of deionized water was added to the low salinity pots (500 mL, 0–0.01ppt). We began saltwater treatments a week after planting. Salinity levels were chosen to represent average salinity conditions for freshwater (0-3ppt) and saline marshes (mean 18ppt, range 8-29ppt) in coastal Louisiana^52^. Salinity was measured with a probe in porewater samples from 14 random pots (7 in low and 7 in high) on six dates throughout the experiment. The high salinity treatment had a mean salinity of 14.75 ppt (standard error 1.6) and the low salinity treatment had an average salinity of 0.19 ppt (SE 0.01). Fertilizer (0.086 g All Purpose Miracle Grow Fertilizer (24% N, 8% P, 16% K) in 200 mL of water) was added to all pots at the onset of the experiment to approximate an aerial rate of 5.6 g N per m^2^ per year, representing a low- nutrient environment^47^.

For the indirect effect treatment, microbial inoculum was collected from a freshwater site (Turtle Cove Research Station, Manchac, LA, average annual salinity of 1.8 ppt) and a saline site (Louisiana Universities Marine Consortium Research Station, Cocodrie, LA, average annual salinity of 18.1 ppt). Analyses of fungal and bacterial soil communities at these sites, along with samples from other sites in freshwater and saline marsh types, showed clustering of microbial communities along a salinity gradient^35^. Variation in community structure between marsh types was greater than variation between sites. Based on these results, we decided that using soil inoculum from one site per marsh type was sufficient. At each of our two sites, soil inoculum was collected (approximately 0-15 cm depth) from 12 locations: four in a *P. australis* monoculture, four in an adjacent native community, and four in a transition area between the invasive and native communities. For each site, to prepare the inoculum, the samples from the 12 locations were pooled, large rocks and rhizomes were removed, small roots were clipped into short sections, and they were homogenized by hand; this yielded two microbial inoculum treatments. For the sterile inoculum treatment, a portion of soil inoculum from both sites was mixed and autoclaved for two cycles at 134 °C at 100kPA for 60 min. All inoculum was stored in a refrigerator for one week and was added to pots on the same day as planting. Inoculum was added to pots at a 1:10 ratio of inoculum to sterile potting medium^44^, so that differences in abiotic soil properties between sites would not impact treatment responses.

Mesocosms were harvested after 3 months. From each pot we collected above and belowground biomass by species. Biomass was dried to a constant weight at 60 °C and weighed.

All data analyses were conducted in R statistical software^54^. To compare the direct and indirect effects of salinity on biomass, we created four separate linear models to test the effects of salinity (low and high), microbial inoculum (freshwater and saline), and their interaction on *P. australis* and native biomass in each community type. In all models, salinity by inoculum interactions were not significant, so they were removed. Pots were not blocked within the greenhouse and thus no random effects were included. For all analyses, total biomass for each pot was adjusted to represent biomass per plant. For *P. australis,* total biomass was divided by six for monoculture pots and by three for mixed pots. For native species, total native biomass was divided by six in native pots, and by three in mixed pots. Linear models were created using the lm function and two-way ANOVA tests were conducted using the anova function. Microbial indirect effects refer to the effect of microbial inoculum, while the salinity addition treatment represents direct effects. The results of these analyses are reported in Table 1.

We conducted a second set of four ANOVA analyses to compare the effects of salinity and microbial treatment on *P. australis* and native biomass, including the sterile inoculum treatment. For these analyses, we created linear models with salinity (low and high), microbial inoculum (sterile, freshwater, and saline), and a salinity x inoculum interaction. If the interaction term was not significant it was removed from the final model. To analyze pairwise differences between treatment means, we ran Tukey post-hoc tests for each community type using the TukeyHSD function in the R stats package. The results of these analyses are displayed in Fig. 1.

We conducted a third set of six ANOVA analyses to compare the effects of salinity and microbial treatment on native biomass separately for each species and community type. For these analyses, we created linear models with salinity (low and high), microbial inoculum (sterile, freshwater, and saline), and a salinity × inoculum interaction. If the interaction term was not significant it was removed from the final model. To analyze pairwise differences between treatment means, we ran Tukey post-hoc tests. The results of these analyses are displayed in Fig. 2.

Bar graphs displaying per plant mean biomass by treatment, species, and community type were created using the package ggplot2^55^.

## Data Availability

The data and R code underlying the analyses in this study are available at: https://github.com/cschro05/MecososmManuscript.
